# Photosynthetic capacity, nutrient status, and growth of maize (*Zea mays* L.) upon MgSO_4_ leaf-application

**DOI:** 10.3389/fpls.2014.00781

**Published:** 2015-01-09

**Authors:** Mareike Jezek, Christoph-Martin Geilfus, Anne Bayer, Karl-Hermann Mühling

**Affiliations:** Faculty of Agricultural and Nutritional Sciences, Institute of Plant Nutrition and Soil Science, Kiel UniversityKiel, Germany

**Keywords:** foliar application, magnesium deficiency, photosynthesis, chlorophyll, cation interaction, *Zea mays* L.

## Abstract

The major plant nutrient magnesium (Mg) is involved in numerous physiological processes and its deficiency can severely reduce the yield and quality of crops. Since Mg availability in soil and uptake into the plant is often limited by unfavorable soil or climatic conditions, application of Mg onto leaves, the site with highest physiological Mg demand, might be a reasonable alternative fertilization strategy. This study aimed to investigate, if MgSO_4_ leaf-application in practically relevant amounts can efficiently alleviate the effects of Mg starvation in maize, namely reduced photosynthesis capacity, disturbed ion homeostasis and growth depression. Results clearly demonstrated that Mg deficiency could be mitigated by MgSO_4_ leaf-application as efficiently as by resupply of MgSO_4_ via the roots in vegetative maize plants. Significant increases in SPAD values and net rate of CO_2_-assimilation as well as enhanced shoot biomass have been achieved. Ion analysis furthermore revealed an improvement of the nutrient status of Mg-deficient plants with regard to [Mg], [K], and [Mn] in distinct organs, thereby reducing the risk of Mn-toxicity at the rootside, which often occurs together with Mg deficiency on acid soils. In conclusion, foliar fertilization with Mg proved to be an efficient strategy to adequately supply maize plants with Mg and might hence be of practical relevance to correct nutrient deficiencies during the growing season.

## INTRODUCTION

The macronutrient magnesium (Mg) is involved in numerous physiological processes during plant growth and development, extending far beyond its well-known function as central atom of chlorophyll. Mg activates more than 300 enzymes, e.g., ribulose-1,5-bisphosphate-carboxylase/oxygenase (RubisCO), glutamine synthetase or glutathione synthase, and therefore participates in the assimilation of carbon, nitrogen, and sulfur, respectively (Marschner, 2012). Furthermore, Mg-binding to ATP is essential for the plasma membrane H^+^-ATPases activity, having Mg-ATP as substrate (Balke and Hodges, 1975). Consequently, phloem loading and partitioning of photoassimilates from source to sink organs depends on Mg availability (Cakmak et al., 1994a,b). Protein biosynthesis cannot take place without Mg, since it connects the subunits of ribosomes (Maathuis, 2009).

Being aware of its impact on plant metabolism, it seems astonishing that Mg has received little attention in agricultural research in the last decades although its deficiency can cause severe reductions in yield and quality of crops (Gransee and Führs, 2013; Neuhaus et al., 2014). Genetic responses to Mg starvation and restoration have been analyzed in the model species *Arabidopsis thaliana* (Hermans et al., 2010a,b), but Cakmak and Yazici (2010) called Mg ‘a forgotten element in crop production.’ The authors consider the deficiency of this nutrient to be a growing problem and limiting factor especially in intensive production systems, consequently also striking human and animal health (Cakmak, 2013).

Magnesium deficiency in plants may occur on soils even under high Mg content of parent soil material. Owing to its unique chemical properties, it is only weakly bound to negatively charged soil particles and is therefore easily replaced by other cations and consequently leached (Maathuis, 2009; Gransee and Führs, 2013). Hence, calcareous soils, acidic soil with excess of protons (H^+^), aluminium (Al) and manganese (Mn), and saline soils with high amounts of sodium (Na) are all prone to Mg deficiency (Mengel and Kirkby, 2001; Gransee and Führs, 2013). Also, elevated rates of NPK-fertilization in high-production systems harbor the risk of Mg deficiency, because strong reciprocal interactions regarding uptake exist among potassium (K) and Mg (Guiet-Bara et al., 2007; Cai et al., 2012). Mg is delivered to the rhizosphere mainly by mass flow (Lynch and St. Clair, 2004) and thus dry soil and low transpiration rates impair Mg uptake additionally. Taking together all these factors that limit Mg availability in soils, an application of Mg directly on leaves where the physiologically Mg demand is highest might be more advantageous than a Mg fertilization of soils with restricted potential of Mg uptake by plant roots. Moreover, such a foliar application allows a precise timing of Mg supply temporally and spatially to satisfy the varying demands of distinct plant organs at different growth stages. Positive effects of Mg foliar fertilization on plant growth have already been described for some crops for example soybean and faba bean (Vrataric et al., 2006; Neuhaus et al., 2013, 2014) but plant responses to foliar fertilizers differ immensely among plant species due to diverse leaf surface properties such as trichomes, cuticula chemistry and cell wall composition (Fernández et al., 2013) and can thus not be generalized. Although it is the most extensively cultivated cereal crop worldwide (Pechanova et al., 2013), the response of maize (*Zea mays* L.) to Mg fertilization is less recognized than for other cereals (Grzebisz, 2013) especially regarding foliar fertilization. To cover the growing worldwide demands, ranging from feed for humans and animals to biofuel production and energy generation, maize cultivation has expanded immensely in the last decade also on marginal soils, which harbor the risk of Mg deficiency (Oikeh et al., 2003).

The aim of this study was to examine whether leaf-application of MgSO_4_ in practically relevant amounts is as efficient as resupply of Mg via the roots in alleviating adverse effects of Mg deficiency in maize, namely low photosynthesis rate, disturbed ionic homeostasis and biomass reduction. A hydroponic cultivation experiment with four distinct experimental groups was conducted in the greenhouse and photosynthesis rate, SPAD values, biomass as well as [Mg], [K], and [Mn] in different organs were determined.

## MATERIALS AND METHODS

### PLANT GROWTH CONDITIONS

**FIGURE 1:**
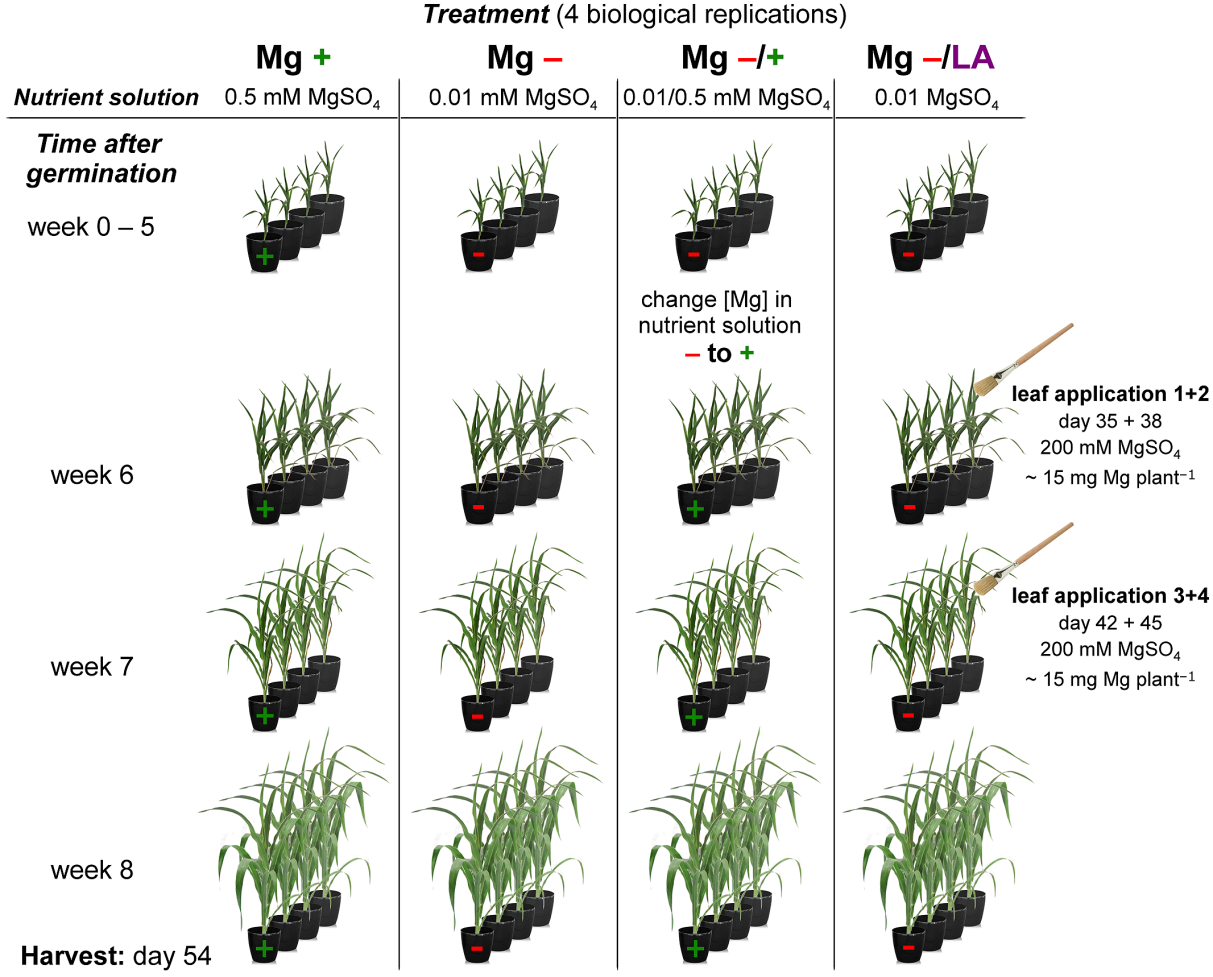
**Schematic view of experimental setup.** Diagram of the hydroponic grown maize plants with four distinct Mg treatments. Mg+, 0.5 mM MgSO_4_ in nutrient solution (NS); Mg-, 0.01 mM MgSO_4_ in NS; Mg–/+, 0.01 mM MgSO_4_ in NS for 5 weeks and 0.5 mM MgSO_4_ in NS for 3 weeks; Mg–/LA, 0.01 mM MgSO_4_ in NS and leaf-application of 200 mM MgSO_4_ for four times. For each treatment group four pots with four maize plants each were installed (only one plant per pot displayed in the simplified diagram). For further details see descriptions in the text (Materials and Methods).

Maize (*Zea mays* L. cv. Susann, Nordsaat Saatzucht, Langenstein, Germany) plants were grown hydroponically in the greenhouse at 20–25°C and 30–50% relative humidity from June–July under natural light regime. Seeds were soaked in 2 mM aerated CaSO_4_ solution for 2 days and germinated in sterile quartz sand, which was kept moistened with 2 mM CaSO_4_. Seven-days-old seedlings were transferred into 9 L plastic vessels (four plants per vessel) with 25% strength nutrient solution (NS), which was raised stepwise to 100% strength within 4 days. The full-strength NS had the following composition: 1.3 mM Ca(NO_3_)_2_, 0.7 mM NH_4_NO_3_, 2.0 mM CaCl_2_, 1.0 mM K_2_SO_4_, 0.2 mM KH_2_PO_4_, 200 μM Fe-EDTA, 5 μM H_3_BO_3_, 2 μM MnSO_4_, 0.5 μM ZnSO_4_, 0.3 μM CuSO_4_, 0.01 μM (NH_4_)_2_Mo_7_O_24_, and it was exchanged weekly. Four different experimental groups with distinct Mg treatments were cultivated in four independent biological replicates, including positive control with sufficient Mg and negative control with severe Mg deficiency (**Figure 1**). Vessels were arranged fully randomized. Plants of the positive control group were supplied with 0.5 mM MgSO_4_ in the NS, whereas the other three groups grew under 0.02 mM MgSO_4_ for 9 days to ensure proper seedling development. In order to induce clear Mg deficiency symptoms, [Mg] was further reduced to 0.01 mM MgSO_4_ thereafter. After a growing period of 5 weeks, the NS [Mg] of one of the three deficiency groups was raised to 0.5 mM MgSO_4_. Another deficiency group was fertilized with Mg over the leaves while still growing in 0.01 mM MgSO_4_ NS. This leaf-application treatment was conducted four times within 10 days by brushing 200 mM MgSO_4_ on the whole plants, namely abaxial and adaxial leaf sides as well as the stems. By this special means the amount of Mg applied which directly reached the plants surface could exactly be quantified. Each plant received 60 mg Mg in total, which would correspond to an application rate of approximately 6 kg ha^-1^ under field conditions. Leaf-application solution contained 0.1% Silwet as wetting agent and treatments took place in the morning to ensure opened stomata and to avoid leaf burnings by high irradiation. Plants were harvested after 54 days of growth.

### SPAD VALUES AND GAS EXCHANGE MEASUREMENTS

Changes in chlorophyll concentration of the 6th leaf were measured non-destructively using a portable chlorophyll meter (SPAD-502, Minolta, Japan) starting at day 26 after germination until harvest. In addition, the ninth leaf was analyzed at harvest. Measurements were performed on two plants per vessel of each of the four biological replicates, four readings per leaf were taken and values were averaged.

Rate of photosynthetic activity was measured regularly starting with the onset of leaf-application on day 35 of the experiment by a portable gas exchange system (LI-6400, LI-COR Biosciences, Lincoln, NE, USA). Measurements were conducted between 10 am and 14 pm on a central 6 cm^2^ leaf segment next to the mid-rib of the sixth leaf. Measurements were taken at two plants of two biological replicates per treatment and values were averaged. Incoming photosynthetic photon flux density provided by a red/blue LED light source (6400-02B, LI-COR Biosciences, Lincoln, NE, USA) amounted to 600 μmol m^-2^ s^-1^ and ambient CO_2_ concentration was adjusted to 400 μmol mol^-1^ by CO_2_ injection (6400-01, LI-COR Biosciences, Lincoln, NE, USA) as described in detail by Geilfus et al. (2011).

### ANALYSIS OF PLANT TISSUE

Fresh weight (FW) of total shoot and several leaves (sixth to eight and youngest leaf) were determined by pooling all four plants per vessel and dividing the values by four.

For mineral analysis, plant tissue was dried at 60°C, ground and 100 mg were digested with 10 mL of 69% HNO_3_ (ROTIPURAN Supra for ICP, 69%) in a 1800 W microwave oven (MARS 6 Xpress; CEM, Matthews, MC, USA) with the following program: 2 min at 100°C, 1 min at 120°C, 20 min at 180°C, 20 min cooling time. Samples were diluted with distilled sterile water (18.2 MΩ cm conductivity) to 100 mL afterward and kept at 4°C until analysis. Concentrations of macro- and micronutrients were quantified using inductively coupled plasma-mass spectrometry (ICP-MS; Agilent Technologies 7700 Series, Böblingen, Germany). The analytical technique was standardized using NCS DC 73350, a certified reference material (leaves of poplar) from the China National Analysis Center for Ion and Steel (Beijing, China). Blank digestions were performed in the same way as for the samples. Every 20 samples, internal macro- and micronutrients standards, as well as reference material samples were analyzed as internal control.

### STATISTICS

Data was statistically analyzed using R statistical package software version 3.0.1 (The R foundation for statistical computing, Vienna, Austria, 2013). Effects of treatments were tested using Student’s *t*-test in case of two experimental groups or with one-way ANOVA and following Bonferroni correction in case of four different groups.

## RESULTS

### SPAD MEASUREMENTS

**FIGURE 2:**
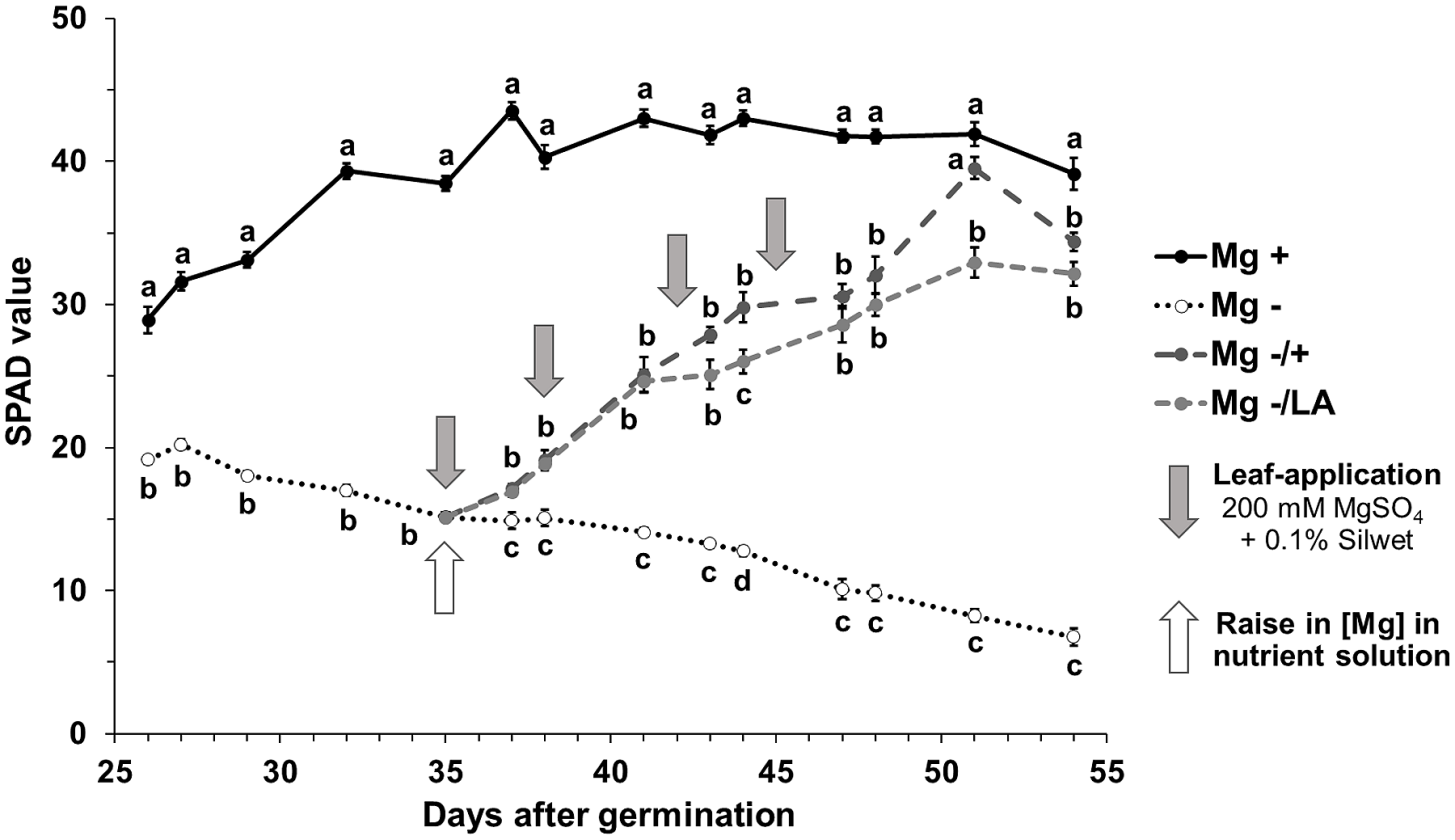
**Course of SPAD values of the sixth leaf over time.** Relative chlorophyll concentration in the sixth leaf assessed by SPAD measurements. Mg+, 0.5 mM MgSO_4_ in NS; Mg-, 0.01 mM MgSO_4_ in NS; Mg–/+, 0.01 mM MgSO_4_ in NS for 5 weeks and 0.5 mM MgSO_4_ in NS for 3 weeks; Mg–/LA, 0.01 mM MgSO_4_ in NS and leaf-application of 200 mM MgSO_4_ for four times. Measurements were made on two plants of each of the four biological replicates per treatment; four individual SPAD values per leaf were averaged. Mean ± SE; arrows show date of leaf-application with 200 mM MgSO_4_; small letters indicate significant differences between treatments for an observation date (ANOVA with Bonferroni adjustment, *p* ≤ 0.05).

The comparison between the Mg sufficient positive control plants (0.5 mM MgSO_4_ in NS) and the Mg deficient negative control plants (0.01 mM MgSO_4_ in NS) revealed that Mg deficiency significantly influenced the chlorophyll concentrations in maize, as assessed by SPAD values (**Figure 2**). While SPAD values raised in leaves of well-nourished positive control plants until day 42 after germination and remained constant henceforth (black line), those of Mg deficient plants decreased until harvest (dotted line). With the onset of Mg resupply to deficient plants on day 35 after germination, SPAD values increased immediately in plants with root- and leaf-resupply to the same degree (light- and dark–gray dashed lines). Both treatments resulted in significantly elevated SPAD values compared to negative control plants after 2 days. At the end of the experiment, SPAD values in the sixth leaf of plants with Mg resupply did not reach the level of the positive control plants, regardless of whether Mg was given to the roots or the leaves. However, SPAD values in the ninth leaf were equal to the well-nourished positive control in both leaf-resupply and root-resupply treatment at harvest time (**Table 1**).

**Table 1:** SPAD values in the ninth leaf at day 54 after germination.

	Treatment	SPAD	SE
Mg+	(Positive control)	37.8^a^	1.0
Mg–	(Negative control)	16.0^b^	0.5
Mg–/+	(Root-resupply)	37.2^a^	0.8
Mg–/LA	(Leaf-application)	35.7^a^	1.5

*Mg+, 0.5 mM MgSO_4_ in nutrient solution (NS); Mg–, 0.01 mM MgSO_4_ in NS; Mg–/+, 0.01 mM MgSO_4_ in NS for 5 weeks and 0.5 mM MgSO_4_ in NS for 3 weeks; Mg–/LA, 0.01 mM MgSO_4_ in NS and leaf-application of 200 mM MgSO_4_ for four times. Measurements were made on two plants of each of the four biological replicates per treatment; four individual SPAD values per leaf were averaged. Mean ± SE; small letters indicate significant differences between treatments (ANOVA with Bonferroni adjustment, *p* ≤ 0.05).*

### PHOTOSYNTHESIS RATE AND TRANSPIRATION

**FIGURE 3:**
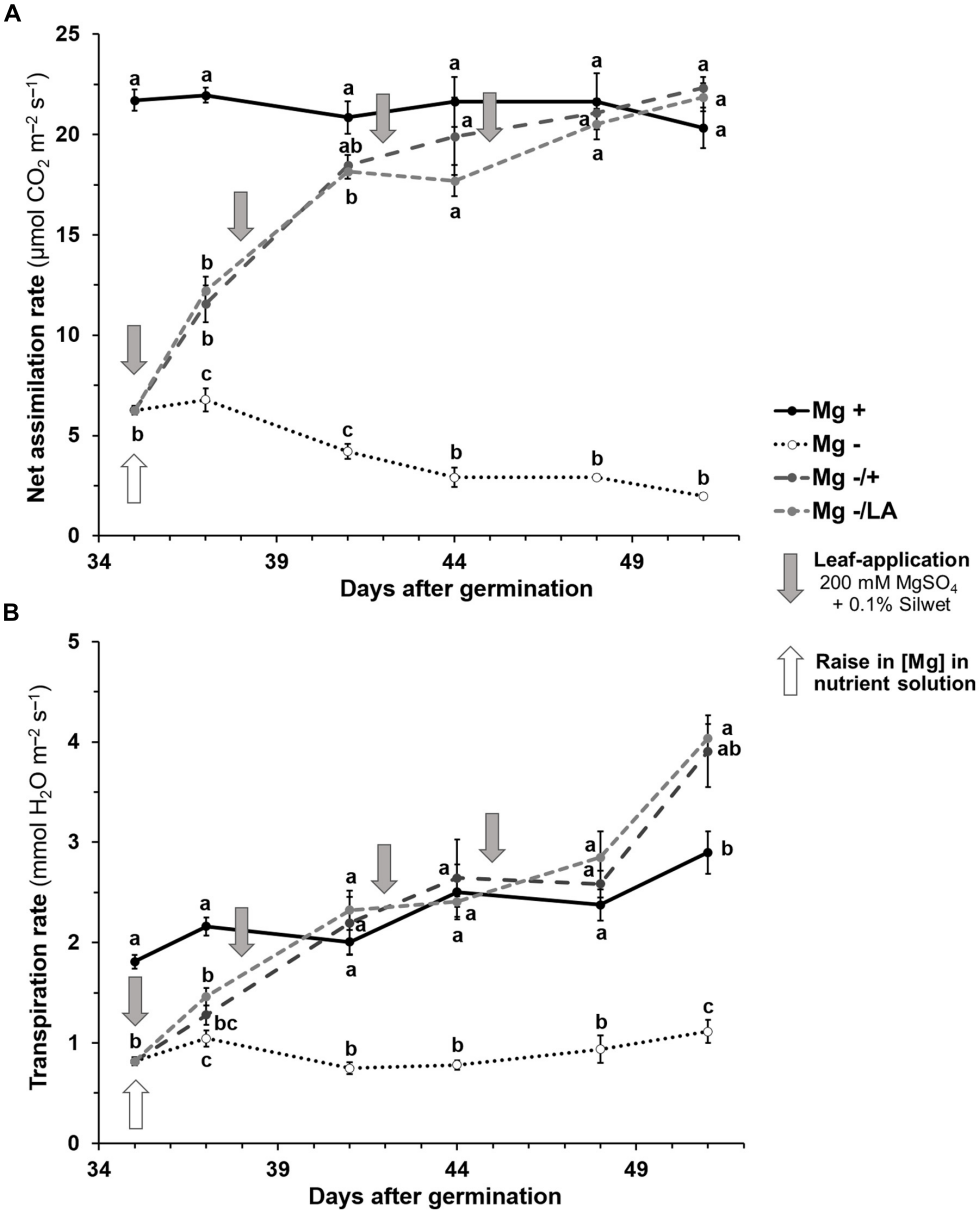
**Course of gas exchange measurements of the sixth leaf over time. (A)** Net CO_2_ assimilation rate (μmol CO_2_ m^-2^ s^-1^) of the sixth leaf. **(B)** Transpiration rate (mmol H_2_O m^-2^ s^-1^) of the sixth leaf. Mg+, 0.5 mM MgSO_4_ in nutrient solution (NS); Mg–, 0.01 mM MgSO_4_ in NS; Mg–/+, 0.01 mM MgSO_4_ in NS for 5 weeks and 0.5 mM MgSO_4_ in NS for 3 weeks; Mg–/LA, 0.01 mM MgSO_4_ in NS and leaf-application of 200 mM MgSO_4_ for four times. Measurements were made on two plants of two biological replicates per treatment. Mean ± SE; arrows show date of leaf-application with 200 mM MgSO_4_; small letters indicate significant differences between treatments for an observation date (ANOVA with Bonferroni adjustment, *p* ≤ 0.05).

Gas exchange measurements revealed significant differences in net CO_2_-assimilation and transpiration rates of the 6th leaf between Mg deficient negative control and well-nourished positive control plants 5 weeks after germination (**Figures 3A,B**). The 6th leaf of well-nourished control plants had stable assimilation rates of about 21 μmol CO_2_ m^-2^ s^-1^ during the course of the experiment (black line) whereas a continuous decrease was observed under Mg deficiency (dotted line) from 6.8 μmol CO_2_ m^-2^ s^-1^ at day 37 to 2.0 CO_2_ m^-2^ s^-1^ at day 51 (dotted line). Transpiration rates of the 6th leaf of Mg deficient plants were significantly reduced compared to well-nourished plants throughout the whole measuring period. Resupply of Mg to the NS (dark-gray dashed line) or via leaf-application (light-gray dashed line) significantly raised both net CO_2_-fixation and transpiration rate within 2 days in comparison to the negative control. Assimilation rates continued to increase until they reached the level of positive control plants 6 days after Mg resupply via the roots and after 9 days, if MgSO_4_ was applied onto leaves, respectively. The latter had been treated with 200 mM MgSO_4_ solution for three times by then, which amounts to 45 mg Mg per plant. Transpiration rates equaled those of positive control plants after 6 days in both treated groups.

### PLANT GROWTH

**FIGURE 4:**
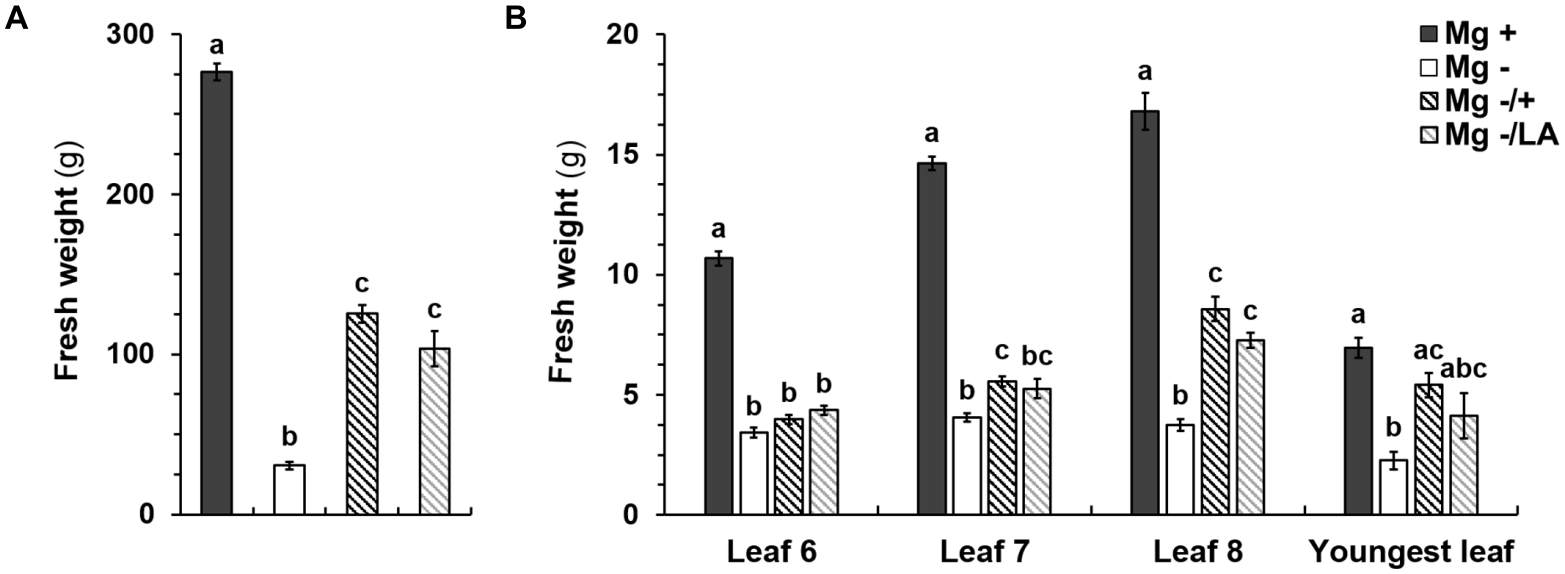
**Fresh weights (FWs) of shoots and single leaves. (A)** FW (g) of shoots and of **(B)** leaves number six, seven, eight, and youngest leaves at harvest (54 days after germination). Mg+, 0.5 mM MgSO_4_ in NS; Mg–, 0.01 mM MgSO_4_ in NS; Mg–/+, 0.01 mM MgSO_4_ in NS for 5 weeks and 0.5 mM MgSO_4_ in NS for 3 weeks; Mg–/LA, 0.01 mM MgSO_4_ in NS and leaf-application of 200 mM MgSO_4_ for four times; mean (*n* = 4 biological replicates) ± SE; small letters indicate significant differences between treatments (ANOVA with Bonferroni adjustment, *p* ≤ 0.05).

Shoot FW of negative control plants was significantly reduced and amounted to 30.5 g shoot^-1^ which accounted for 11.0% of adequately nourished positive control plants (**Figure 4A**). This reduction in FW of negative controls was also apparent in leaves number six, seven, eight, and in the youngest leaves (**Figure 4B**). Mg supply after 5 weeks of deficiency significantly raised shoot FW to the same extent regardless of whether it was given to the NS or applied onto leaves (**Figure 4A**). Mean FW were 125.3 g shoot^-1^ for root-resupply and 103.4 g shoot^-1^ in plants with MgSO_4_ leaf-application. Resupply of Mg increased FW of the eighth leaf to the largest extent (**Figure 4B**). In comparison to Mg-deficient plants, no increase in FW was observed in leaf number six upon Mg resupply via leaves or the NS. Mg addition to the NS raised the FW of leaf number seven and the youngest leaf compared to the negative control plants. Upon MgSO_4_ leaf-application FW of these leaves were not significantly different from neither the negative control nor the plants with Mg-resupply via the roots.

### Mg, K AND Mn CONCENTRATIONS IN DISTINCT PLANT ORGANS

**FIGURE 5:**
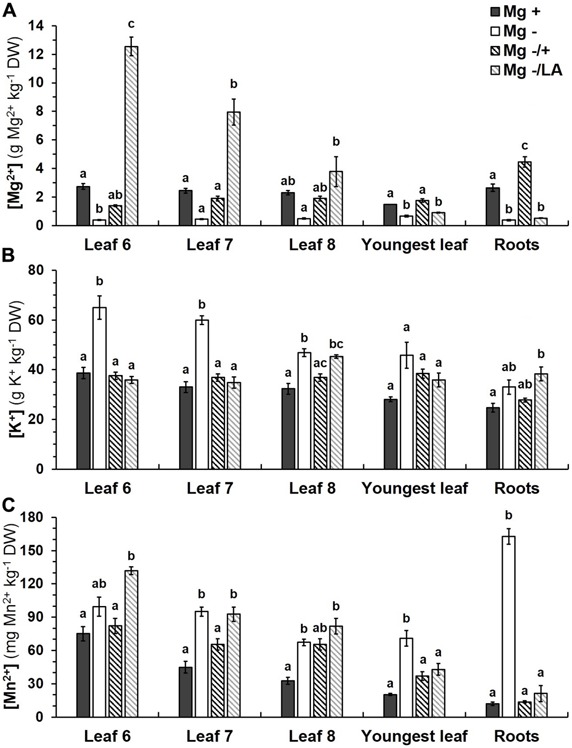
**Ion concentrations in various leaves and roots. (A)** [Mg], **(B)** [K], and **(C)** [Mn] were analyzed in different tissues at harvest (54 days after germination). Mg+, 0.5 mM MgSO_4_ in NS; Mg–, 0.01 mM MgSO_4_ in NS; Mg–/+, 0.01 mM MgSO_4_ in NS for 5 weeks and 0.5 mM MgSO_4_ in NS for 3 weeks; Mg–/LA, 0.01 mM MgSO_4_ in NS and leaf-application of 200 mM MgSO_4_ for four times; mean (*n* = 4 biological replicates ± SE; small letters indicate significant differences between treatments (ANOVA with Bonferroni adjustment, *p* ≤ 0.05).

Positive control plants cultivated in NS with 0.5 mM MgSO_4_ showed [Mg] between 1.5 g Mg kg^-1^ dry weight (DW) in youngest and 2.7 g Mg kg^-1^ DW in oldest investigated leaves; root tissue contained 2.7 g Mg kg^-1^ DW (**Figure 5A**). Severe Mg starvation affected [Mg] significantly in the sixth leaf, in the youngest leaves and in root tissue. Addition of 0.5 mM MgSO_4_ to the NS after 5 weeks of Mg deficiency significantly raised [Mg] in all leaves and, predominantly, in the roots, which contained almost 70% more Mg in comparison to positive control plants. Resupply of Mg through leaf application resulted in a prominent increase of [Mg] in basal leaves. Leaves number six to eight contained 3.8 up to 12.6 g Mg kg^-1^ DW, increasing with decreasing leaf number. Youngest leaves and roots, in contrast, were not affected and did not have a significantly higher [Mg] than Mg deficient negative control plants.

The dose and method of Mg nutrition also influenced [K] in the plant tissues (**Figure 5B**). Growth in NS with 0.01 mM MgSO_4_ elevated [K] significantly in leaves number six to eight, but not in youngest leaves and roots. With 46.9 to 65.0 g K kg^-1^ DW, [K] in basal leaves of negative control plants were up to 1.8 times as high as in positive control plants, which contained 32.3 to 38.7 g K kg^-1^ DW. Re-nutrition with MgSO_4_ via roots significantly reduced [K] in whole shoots and roots to levels of control plants, whereas [K] in the eighth leaf and roots remained elevated, if MgSO_4_ leaf-application was performed.

Magnesium deficient negative control plants showed high [Mn] not only in leaves, but predominantly in roots. With 162.8 mg Mn kg^-1^ DW they contained 13.4 times more Mn than positive control plants (12.1 mg Mn kg^-1^ DW; **Figure 5C**). Increasing the MgSO_4_ concentration in the NS led to a significant decrease of [Mn] in all examined plant parts to levels of the positive control group. Leaf-application of MgSO_4_, by contrast, only reduced [Mn] significantly in youngest leaves and roots, whereas they remained as high as in negative control plants in the older leaves.

## DISCUSSION

Magnesium deficiency is considered to be a growing problem throughout agriculture and it occurs on diverse soil types (Mengel and Kirkby, 2001). Since Mg availability in soil and uptake into the plant often is limited foliar fertilization instead of Mg addition to the soil seems reasonable for meeting the crop’s demands. Therefore, it was questioned, if foliarly applied Mg could alleviate Mg deficiency syndromes in vegetative maize plants as fast and efficient as a resupply via the roots. The amount of foliarly applied Mg was exactly quantified and amounted to 60 mg Mg per plant in total, which corresponds to a practically relevant application rate of approximately 6 kg Mg ha^-1^. Several physiological reactions of maize plants under severe Mg starvation and subsequent resupply of Mg via leaf- or root-fertilization were investigated by hydroponic cultivation.

Negative control plants grown in 0.01 mM MgSO_4_ showed strong reductions in photosynthesis and biomass, which is in accordance with several studies on other crops (Gerendás and Führs, 2013; Neuhaus et al., 2014). The negative effects on such yield-relevant physiological processes once again illustrate the importance of Mg as a plant macronutrient and underline the need for developing adequate Mg application strategies to ensure high plant productivity.

### LEAF-APPLICATION OF MgSO4 RAPIDLY INCREASES [Mg] IN Mg DEFICIENT MAIZE

**FIGURE 6:**
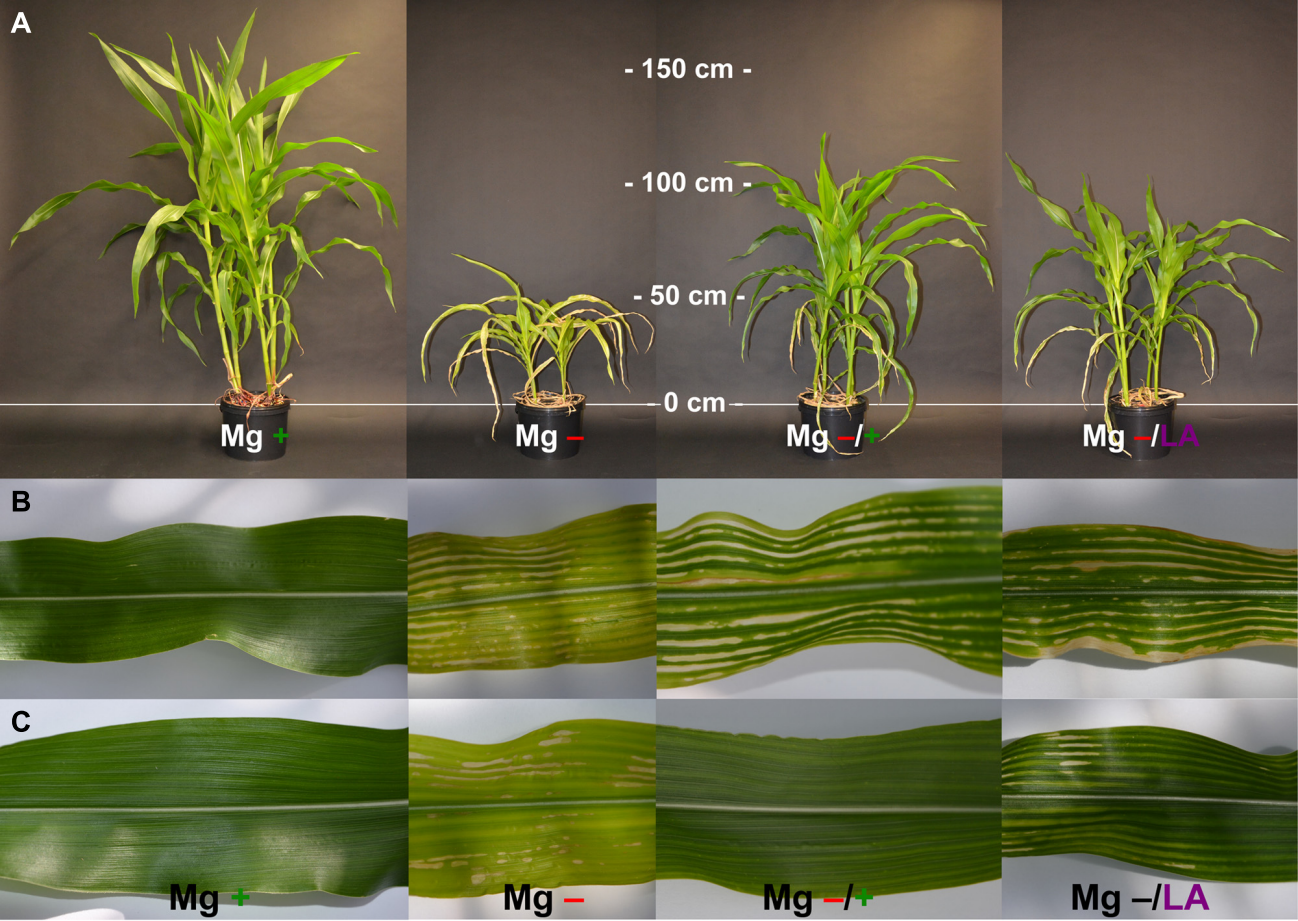
**Visual symptoms of Mg deficiency and effects of Mg resupply. (A)** Representative plants at harvest time (54 days after germination); **(B,C)** representative leaves of the sixth and seventh leaf respectively at day 44 after germination; plants with MgSO_4_ leaf-application had received three of four application-treatments by then. Mg+, 0.5 mM MgSO_4_ in NS; Mg–, 0.01 mM MgSO_4_ in NS; Mg–/+, 0.01 mM MgSO_4_ in NS for 5 weeks and 0.5 mM MgSO_4_ in NS for 3 weeks; Mg–/LA, 0.01 mM MgSO_4_ in NS and leaf-application of 200 mM MgSO_4_ for four times.

Bergmann (1983) states [Mg] in fully expanded maize leaves to range from 2.5 to 6.0 g kg^-1^ DW. According to that threshold, positive control plants were sufficiently supplied with Mg whereas Mg deficient negative controls were growing under severe malnutrition (**Figure 6A**). Increasing [Mg] in the NS after 5 weeks of Mg restriction led to elevated [Mg] especially in newly developed leaves and to minor extent in those, which had already been built under deficiency conditions (**Figure 5A**). Furthermore, [Mg] in roots markedly increased, which might indicate elevated uptake after a time of nutrient deprivation. This would be in agreement with results from Cai et al. (2012), who found a putative Mg transporter gene to be up-regulated under Mg deficiency in rice. Nevertheless, our data do not reveal Mg uptake into the symplast of root cells since cation concentrations of the whole tissue has been analyzed. Leaf-application of 200 mM MgSO_4_ remarkably increased [Mg] in leaves that had directly been treated by foliar application. Declining [Mg] in the upper leaves are attributable to the number of foliar application treatments. The sixth leaf was treated four times with MgSO_4_ solution whereas leaf number eight only received one application. It is surprising that MgSO_4_ leaf-application did not affect [Mg] in roots and youngest leaves, which did not receive MgSO_4_ application, since Mg is known to be a highly phloem-mobile cation, which can be translocated within the plant to sites of high nutrient demand (Steucek and Koontz, 1970; White and Broadley, 2009). However, the Mg demand of roots is relatively low, given its primary role in chloroplasts (Maathuis, 2009) and [Mg] in youngest leaves obviously were high enough to significantly increase chlorophyll concentrations in youngest leaves (**Table 1**), as SPAD values were not different from well-nourished control plants at harvest.

### MAGNESIUM RESUPPLY VIA LEAVES RAPIDLY ENHANCES PHOTOSYNTHETIC PARAMETERS IN Mg DEFICIENT MAIZE

Chlorophyll concentrations estimated by SPAD measurements were markedly reduced under Mg deficiency (**Figure 2**, dotted line) and leaves showed deficiency symptoms being typical in cereals like interveinal yellowing, chlorosis, and necrosis (**Figures 6B,C**). Mg resupply elevated SPAD values significantly already within 2 days and application of 200 mM MgSO_4_ to maize leaves turned out to be as efficient in raising SPAD values as addition of 0.5 mM MgSO_4_ to the roots (**Figure 2**, dark-gray and light-gray dashed lines, respectively). Even one single leaf application treatment and hence 15 mg Mg per plant was sufficient for triggering significant effects. The efficient uptake of MgSO_4_ solution into maize leaves can be ascribed to their amphistomatical anatomy and their wettability at both leaf sides (Urrego-Pereira et al., 2013). Raise in chlorophyll concentration by Mg foliar-application has also been shown for faba bean, oregano, and soybean before (Dordas, 2009; Teklic et al., 2009; Neuhaus et al., 2013). Mg not only serves as central atom of chlorophyll molecules but is also necessary in chlorophyll biosynthesis, activating Mg-chelatase, a complex enzyme which catalyzes the insertion of Mg into a chlorophyll precursor molecule (Reid and Hunter, 2004; Masuda, 2008). The high biological relevance of Mg for chlorophyll production hence explains the pronounced re-greening of all leaves already within a few days after Mg resupply (**Figures 6B,C**). Not only leaves that were directly treated with MgSO_4_ showed higher SPAD values compared to Mg-deficient control plants, but the effects of the leaf-application also became visible in the youngest, untreated leaves, where SPAD values were not significantly different from control plants or plants with root resupply at harvest time (**Table 1**). This suggests the uptake of the applied Mg into the cytosol and is furthermore indicative for the systemic effect of MgSO_4_ leaf-application in distant, untreated plant parts.

Increasing chlorophyll level assessed by SPAD measurements were certainly one reason why CO_2_-assimilation in the re-supplied treatments equaled to the level of the positive control plants after Mg had been restored via leaves or roots (**Figure 3A**). Similar to the SPAD values, the net CO_2_-fixation rate responded rapidly to Mg addition. Besides being the central atom of chlorophyll, Mg is furthermore involved in several other processes related to photophosphorylation and CO_2_-fixation (Lin and Nobel, 1971; Verbruggen and Hermans, 2013). For instance, Mg acts in charge balancing during the establishment of the proton motive force and activates several enzymes involved in photosynthesis, including RubisCO (Marschner, 2012). Also the transpiration rate rose upon Mg addition (**Figure 3B**), as was earlier observed in rice plants under Mg deficiency (Kobayashi et al., 2012). According to the authors this might be caused by a relationship between the ABA signaling network and Mg deficiency. The results show that the adverse effects of Mg deficiency on plant metabolism can be amended by Mg leaf-application as efficiently as by root resupply in maize plants, since no considerable differences between both treatments were detected.

Magnesium deficient maize plants were sufficiently supplied with sulfur through the NS (1 mM sulfur) and showed no symptoms of sulfur deficiency throughout the whole growth period (**Figures 6B,C**). Hence, the described effects of MgSO_4_ leaf-application on physiological processes can be attributed to the influence of Mg. Not only the added Mg directly improved photosynthetic processes through its participation in chlorophyll formation and enzyme activation, but a contribution of secondary effects is likely. To these belong for example, the restoration of ionic homeostasis especially regarding [Mn] in leaves because Mn stress was found to negatively impact the abundance of chloroplastic proteins important for CO_2_ fixation (Führs et al., 2008; Millaleo et al., 2013).

### MAGNESIUM FOLIAR APPLICATION DECREASES THE RELATIVE UPTAKE OF OTHER NUTRIENTS AT THE ROOTSIDE

Ion analysis revealed that the Mg resupply not only affects [Mg] but also other cations such as K and Mn (**Figures 5B,C**). It has frequently been observed that variations in one nutrient is compensated by changing uptake of other cations instead (Morgan and Jackson, 1976; Mengel and Kirkby, 2001). Cations are in competition for apoplastic binding sites, e.g., negatively charged pectins, as well as for further uptake for example through non-selective cation channels (NSCC), which can be passed by both monovalent and divalent cations (Shabala and Hariadi, 2005). Hence, reciprocal effects among cations with respect to uptake into and distribution within the plant exist and antagonistic interactions of Mg, Ca, and K have been demonstrated for example in onion (Kleiber et al., 2012). Interestingly, amelioration of Mg deficiency by root- or leaf-application reduced [K] and [Mn] in distinct plant tissues to a varying extent.

### ELEVATED [K] UNDER Mg DEFICIENCY ARE DECREASED BY LEAF-APPLICATION OF Mg IN MAIZE SHOOTS

Potassium concentrations, normally ranging from 30 to 45 g kg^-1^ DW in maize leaves (Bergmann, 1983), were found to be unusually high in Mg deficient plants, especially in older leaves (**Figure 5B**). These results seem plausible since K is a strong competitor for other cations due to its efficient uptake systems (Mengel and Kirkby, 2001) and are in accordance with results from Cai et al. (2012), who measured much higher K concentrations in rice shoots under Mg starvation and a moderate up-regulation of the expression of a high affinity K transporter gene. Such a hyper-accumulation of K with a subsequent growth-reduction has also been shown for plants, which were genetically restricted in their ability to store Mg in the vacuole (Gilliham et al., 2011). Elevated [K] under Mg deficiency might not only be explained by increased relative uptake, but also decreased export out of leaves into physiological sinks, since this process depends on ATPase activity and is known to be disturbed under Mg deficiency (Cakmak et al., 1994a,b).

Potassium concentrations decreased to the level of control plants upon resupply of Mg to the NS both in shoots and roots of maize, illustrating again the competitive interaction between these cations. Decreased K flux through NSCC with increasing [Mg] has also been detected in broad bean (Shabala and Hariadi, 2005). In contrast to Mg resupply via roots, [K] remained high in roots, if MgSO_4_ was foliarly applied to Mg deficient plants. This would be expected due to absence of Mg as a competitor in uptake at the rootside and the low [Mg] in the root tissue (**Figure 5A**). Nevertheless foliar treatment successfully lowered [K] in most investigated leaves significantly, not only in expanding tissue but also in mature leaves which have been built under deficient conditions.

### LEAF-APPLICATION OF MgSO_4_ REDUCES HIGH Mn CONCENTRATIONS IN Mg DEFICIENT MAIZE ROOTS

Manganese is an essential micronutrient for plants and a major rival for Mg respecting uptake and transport due to their similar ionic radius and biochemical properties (Marschner, 2012). It occurs in different oxidation states and therefore plays a prominent role in redox processes, but can easily become phytotoxic if taken up in excess, which appears for example on soil with low pH or high redox potential (Kopittke et al., 2013). Effects of the competitive relationship between Mg and Mn vary between plant species, type of cultivation and nutrient concentrations in the medium. For example, elevated [Mn] in leaves of Mg deficient potato plants and wheat were measured (Cao and Tibbitts, 1992; Chatterjee et al., 1994), whereas the competitive effect of Mg on the uptake of Mn was low in rapeseed (*Brassica napus*) and hardly existed in lucerne (*Medicago sativa*; Löhnis, 1960).

Maize grown under Mg deficiency showed high [Mn] not only in leaves but especially in roots (**Figure 5C**). This might partially be caused by the absence of Mg as a competitor and subsequently increased Mn uptake at the rootside. However, a dilution effect caused by impaired root growth under Mg deficiency, which has been observed in bean plants for example (Cakmak et al., 1994b), might have contributed to markedly increased [Mn] in root tissue. In fact, the total amounts of accumulated Mn were the same for Mg deficient and well-supplied control plants (data not shown). Mn is commonly known to be easily transported from roots to above-ground plant organs (Horst, 1983), but excess accumulation of Mn in roots has been observed in cultivars of grape (Mou et al., 2011) and certain maize varieties (Stoyanova et al., 2009). Since Mn is easily transported from roots to the shoot in the xylem (Page and Feller, 2005), strong depression of transpiration under Mg deficiency (**Figure 3B**) might have further added the high [Mn] in roots. Impaired nutrient transport activities upon Mg deficiency have also been found in rice and have been explained by reduced transpiration (Kobayashi et al., 2012). Mn concentrations in roots of Mg deficient plants had almost reached toxic levels. The normal range of [Mn] is stated to be 40 to 150 mg Mn kg^-1^ DW in maize shoots (Bergmann, 1983) and the critical toxicity concentration amounts to approximately 200 mg Mn kg^-1^ DW (Marschner, 2012), but clear differences in Mn tolerance exist among maize varieties (Stoyanova et al., 2009). Thus maize grown under severe Mg deficiency might already have experienced Mn toxicity at the rootside, being accountable for the observed shedding of fine roots during the experiment (data not shown). Such a loss of roots and reduced root growth in connection with increased [Mn] in root tissue has also been found in grape (*Vitis vinifera*) and was explained partially by oxidative stress leading to cellular damage (Mou et al., 2011). The phenomenon of root abscission due to oxidative stress has also been described for plants growing under heavy metal stress (Razinger et al., 2008; Xing et al., 2010).

Magnesium resupply to the NS successfully lowered [Mn] in both leaves and roots to the level of control plants, whereas Mg leaf-application reduced [Mn] only in youngest leaves and roots (**Figure 5C**). Reduction of [Mn] by increasing Mg supply to the roots has already been demonstrated (Löhnis, 1960; Elamin and Wilcox, 1985; Goss and Carvalho, 1992) but, to our knowledge, it was never shown before that this effect could at least partially be realized by Mg leaf-application. This finding is particularly interesting, since Mg deficiency often occurs on acid soils (Mengel and Kirkby, 2001), where an elevated risk of Mn toxicity exists due to reduction of MnO_2_ to Mn, the plant-available form of this micronutrient. Consequently, Mg deficient plants growing in soil with low pH are especially prone to Mn toxicity. Lowering this risk by Mg addition to the soil has already been recommended (Löhnis, 1960) but leaf-application is much more favorable, since Mg is easily leached out in acid soils.

## CONCLUSION

Magnesium deficiency severely compromises physiological efficiency of maize plants. Foliar fertilization of maize plants with MgSO_4_ turned to be an efficient means of Mg supply under severe Mg deficiency. Leaf-application of Mg proved to be as efficient as Mg resupply via the roots in ameliorating adverse effects of Mg deficiency, namely reduced photosynthetic capacity, high concentrations of other cations especially Mn in different plant tissues and reduced biomass. It might hence be of practical relevance to correct nutrient deficiencies during the growing season especially if Mg availability via root-uptake is limited due to poor soil properties or climatic conditions.

## Conflict of Interest Statement

The authors declare that the research was conducted in the absence of any commercial or financial relationships that could be construed as a potential conflict of interest.
